# Validation of sociocultural attitudes towards appearance questionnaire and its associations with body-related outcomes and eating disorders among Chinese adolescents

**DOI:** 10.3389/fpsyt.2023.1088769

**Published:** 2023-03-13

**Authors:** Houyi Huang, Zhongting Liu, Haoran Xiong, Fabian Herold, Jin Kuang, Erle Chen, Alyx Taylor, Albert Yeung, Jing Sun, Md M. Hossain, Arthur Kramer, Tianyou Guo, Liye Zou

**Affiliations:** ^1^Body-Brain-Mind Laboratory, The Shenzhen Humanities & Social Sciences Key Research Bases of the Center for Mental Health, School of Psychology, Shenzhen University, Shenzhen, China; ^2^Research Group Degenerative and Chronic Diseases, Movement, Faculty of Health Sciences Brandenburg, University of Potsdam, Potsdam, Germany; ^3^Shenzhen College of International Education, Shenzhen, China; ^4^AECC University College, School of Rehabilitation, Sport and Psychology, Bournemouth, United Kingdom; ^5^Department of Psychiatry, Harvard Medical School, Boston, MA, United States; ^6^Depression Clinical and Research Program, Massachusetts General Hospital, Boston, MA, United States; ^7^School of Medicine and Dentistry and Menzies Health Institute Queensland, Griffith University, Birtinya, QLD, Australia; ^8^Department of Decision and Information Sciences, C.T. Bauer College of Business, University of Houston, Houston, TX, United States; ^9^Department of Health Systems and Population Health Sciences, Tilman J. Fertitta Family College of Medicine, University of Houston, Houston, TX, United States; ^10^Center for Cognitive & Brain Health, Northeastern University, Boston, MA, United States; ^11^Beckman Institute, University of Illinois, Champaign, IL, United States

**Keywords:** sociocultural attitudes towards appearance questionnaire-4R, eating disorders, Chinese adolescents, body dissatisfaction, validation

## Abstract

**Introduction:**

The Sociocultural Attitudes Towards Appearance Questionnaire-4 Revised (SATAQ-4R) has been widely used in Western countries to link body appearance that is related to eating disorders and body dissatisfaction being commonly reported by adolescents. However, a comprehensive psychometric validation of the SATAQ-4R in Chinese adolescent samples is still lacking. To this end, the aim of the current study was to validate the gender-appropriate SATAQ-4R in a sample of Chinese adolescents, following by an investigation of its associations with body-related outcomes and eating disorder symptoms.

**Methods:**

Two gender-specific studies were conducted to examine the psychometric properties of the SATAQ-4R-Female and SATAQ-4R-Male respectively among adolescent girls (Study1, *N*=344, with 73 participants at retest) and boys (Study2, *N*=335, with 64 participants at retest). Confirmatory factor analysis was employed to examine the factor structure and their test-retest reliability, the internal consistency and convergent validity were subsequently examined.

**Results:**

For the SATAQ-4R-Females, the seven-factor model has a reasonable fit, with Chi-square =1112.769 (*p* < 0.001), CFI = 0.91, RMSEA = 0.071, SRMR = 0.067. For the SATAR-4R-Males, an acceptable seven-factor model with Chi-square = 982.92 (*p*<0.001), CFI = 0.91, RMSEA = 0.08, SRMR= 0.06 was observed. With respect to test-retest reliability, the internal consistency for 7 subscales was rated as good (Cronbach’s alpha =0.74 to 0.95) among female adolescents, likewise the internal consistency of the seven subscales was also rated as good (Cronbach’s alpha =0.70 to 0.96) among male participants. Good convergent validity was observed, reflected by associations of the subscales of the gender-specific SATAQ-4R with muscularity-related attitude, body image-acceptance, body appearance, perceived stress level, symptoms of eating disorder and self-esteem.

**Discussion:**

For women and men, the original 7-factor structure was validated among Chinese adolescents, internal reliability coefficients for the seven subscale scores were good and test-retest reliability was acceptable. Our results also confirmed the convergent validity of the two different gender-appropriate scales.

## Introduction

Body dissatisfaction refers to persistent negative emotions and thoughts about his or her own body weight and physical appearance (1–4). Such body-related misconception is relatively common among Chinese adolescents, as a study reported that 72.7% of Chinese middle-school students perceived themselves as fat, even though only 5.3% of them were objectively overweight (5). Another study observed that 47.2% and 56.5% of Chinese boys and girls, respectively, reported body dissatisfaction and were over-concerned about their body size (6). Of note, the occurrence of body dissatisfaction has been linked to a higher risk of developing disordered eating patterns (7, 8), which is perhaps caused by excessive preoccupations with respect to the individual physical appearance (9). In addition, Chinese adolescents who reported a higher level of shape- or weight-related concerns or dissatisfaction were linked to lower self-esteem and higher levels of depressive symptoms (10, 11), which, in turn, resulted in the increased prevalence of suicidal thought (12). As a result, body dissatisfaction does not only negatively influence adolescent well-being (13), but also impacts health and social systems due to the collective burden of eating disorders and other comorbidities associated with body dissatisfaction (14). Based on a wide range of negative consequences of body dissatisfaction among the affected individuals and communities, there is an urgent need to examine factors that contribute to shape- or body-related perceptions (i.e., body dissatisfaction).

To assess the influence of sociocultural attitudes on the perception of adolescents’ physical appearance, the Sociocultural Attitudes Towards Appearance Questionnaire (SATAQ) was developed and has been widely used to investigate relationships between eating disorders and body image concerns. Since its first publication in 1997, the SATAQ has gone through several revisions to account for the shift in aesthetic standards and appearance ideals. Of note, the SATAQ-4 is the first version that includes both males and females (15). The psychometric properties of the SATAQ have been confirmed in multiple countries, including eastern countries such as Japan whose culture, at least partly, resembles Chinese culture. Despite SATAQ-4 providing valid measures of the internalization of appearance ideals and appearance-associated pressures derived from peers, family, and media, there are some conceptual limitations (e.g., items about athleticism represented not only muscularity but also some physical competence such as coordination and agility, resulting in ambiguity in subscale score interpretation) that required further adjustment to provide a more precise and accurate assessment. Therefore, a newly adapted SATAQ-4R was developed and its advantages have been documented in a previous study (16). The SATAQ-4R has been applied in a study (17), focusing on the validation of the Chinese version of the Acceptance of Cosmetic Surgery Scale (ACSS), in which six factors for male adults (Internalization of Muscularity, Internalization of Thinness, Internalization of General Attractiveness, Pressure from Family, Pressure from Peers and Significant Others, and Pressure from Media) and four factors for female adults (Internalization of Muscularity, Internalization of Thinness and General Attractiveness, Pressure from media) were supported. However, given the main focus of the above-mentioned study, a comprehensive psychometric validation of the SATAQ-4R in Chinese samples (e.g., adolescents) is still lacking. As the SATAQ-4R has not been appropriately validated in Chinese adolescents, further studies are needed since adolescence is a unique stage in individual development.

To summarize, given the high prevalence of body dissatisfaction (i.e., misconception about physical appearance) and its significant impact on Chinese adolescents’ physical and mental health, effective tools to measure theoretical constructs that can explain factors (e.g., internalization of ideal appearance and sociocultural pressure) contributing to the development of body dissatisfaction are urgently required. Considering that there are gender-related differences in the field of body dissatisfaction, the SATAQ-4R which considers and accounts for these gender-related differences, is a more suitable instrument than previous versions of the questionnaire. Thus, the SATAQ-4R can be helpful for further research aiming to better understand the complex relationships between, for instance, disordered eating and body dissatisfaction. Thus, in this study, we translated and validated the Chinese version of the SATAQ-4R in a sample of Chinese adolescent girls and boys.

The aim of the current study was to validate the SATAQ-4R in a sample of Chinese adolescents. Since the seven-factor model has been confirmed in different cultures (18), the original seven-factor model was examined in the current study. Based on previous studies, we expected the internalization of a thin ideal, internalization of muscularity ideal, and the internalization of general appearance would have a medium to large correlation with the body image acceptance, drive for muscularity, and body-esteem, respectively. In addition, we expected the pressure subscales would demonstrate a medium to large correlation with the perceived stress scale. Moreover, we expected that all the SATAQ-4R subscales demonstrate a medium to large correlation with the eating disorder symptoms and a small to medium correlation with self-esteem. In addition, as the SATAQ-4R is an instrument that is based on sociocultural theory and was previously created and validated mostly within western culture, we expected a lower fit for Chinese adolescents due to cultural differences.

## Methods

### Study participants

The participants of the current study were recruited from 3 high schools located in South China and data collection occurred from July 18th to August 5th, 2022. Of note, before data collection, a meeting with school counselors was conducted to ensure that students: (1) were able to understand the questionnaires; (2) had no psychiatric disorder like an eating disorder; (3) were not physically disabled or suffered from other types of physical illness that negatively affect his or her perception about his or her physical condition or body image. Given the nature of SATAQ-4R, female and male participants were asked to complete two respective scales with a different number of items (see Subsection of “Measures”), which generated gender-based samples for data analysis. For the pretest, the female sample consisted of 344 Chinese high school girls aged 13–18 years (15.9 ± 0.49 years; 20.35 ± 3.47 kg/m^2^), while the male sample consisted of 335 Chinese high school boys aged 15–18 years (16.04 ± 0.5 years; 21.02 ± 3.45 kg/m^2^). For a 1-week re-test, 73 female participants (15.88 ± 0.43 years; 20.21 ± 2.76 kg/m^2^) and 64 male participants (15.98 ± 0.45 years; 20.85 ± 3.83 kg/m^2^) volunteered to complete the SATAQ-4R, respectively. Demographic information is presented in Table 1.

**Table 1:** Demographic information for all samples.

	Pretest female sample (*N* = 344)	Test–retest female sample (*N* = 73)	Pre-test male sample (*N* = 335)	Test–retest male sample (*N* = 64)	*p*
Age, years	15.90 (0.49)	15.88 (0.43)	16.04 (0.50)	15.98 (0.45)	<0.01
Height, cm	162 (5.33)	162.43 (4.96)	175 (5.77)	175.37 (5.02)	<0.01
Weight, kg	53.94 (9.64)	53.35 (7.94)	64.42 (11.37)	64.15 (12.32)	<0.01
BMI, kg/m^2^	20.35 (3.47)	20.21 (2.76)	21.02 (3.45)	20.85 (3.83)	<0.05
Only child n (%)	82 (23.84%)	12 (23.08%)	90 (26.87%)	16 (25%)	0.37
Level of education (mother/father)					0.40/0.59
Primary school	17 (4.94%)/7 (2.03%)	2 (3.85%)/0 (0%)	23 (6.87%)/14 (4.18%)	2 (3.13%)/1 (1.56%)	
Secondary school	86 (25%)/67 (19.48%)	12 (23.08%)/10 (19.23%)	82 (24.48%)/59 (17.61%)	17 (26.56%)/8 (12.50%)	
High school	97 (28.2%)/106 (30.81%)	15 (28.85%)/13 (25%)	113 (33.73%)/119 (35.52%)	24 (37.50%)/26 (40.63%)	
Undergraduate degree	136 (39.53%)/150 (43.60%)	23 (44.23%)/27 (51.92%)	103 (30.75%)/132 (39.40%)	18 (28.13%)/27 (42.19%)	
Postgraduate & above	8 (2.33%)/14 (4.07%)	0 (0%)/2 (3.85%)	14 (4.18%)/11 (3.28%)	3 (4.69%)/2 (3.13%)	

BMI = body-mass-index.

**Table 2:** Means and intercorrelations between the Chinese SATAQ-4R Female Subscales (*N* = 344).

	Mean (SD)	1	2	3	4	5	6	7
Internalization: Thin/Low Body Fat	3.10 (0.83)							
Internalization: Muscular	2.41 (0.75)	0.26**						
Internalization: General attractiveness	3.69 (0.64)	0.55**	0.07					
Pressures: Family	2.52 (0.99)	0.40**	0.17**	0.16**				
Pressures: Peers	2.90 (0.94)	0.51**	0.13*	0.39**	0.56**			
Pressures: Significant others	2.47 (1.02)	0.51**	0.18**	0.37**	0.61**	0.68**		
Pressures: Media	2.77 (1.13)	0.46**	0.12*	0.43**	0.45**	0.61**	0.59**	

SD = standard deviation, * = *p* < 0.05, ** = *p* < 0.01.

### Procedure

In the context of this study, following the standard procedures in our previous validation studies (19–21), the SATAQ-4R was translated into Chinese and back-translated into English. Prior to this translation, the authors of the original SATAQ-4R had granted their permission to do so. Specifically, two native Chinese speakers translated the SATAQ-4R items from English into the Chinese language. This step was followed by a meeting with the leading author (L.Y.Z) and 3 graduate students who were fluent in English, which generated a Chinese-language scale. To ensure the accuracy of the translated scale, a back translation was conducted by another Chinese-English bilingual scholar who was not involved in the study. The back-translated English version and the original version were carefully compared to create a prefinal Chinese version, which captured the complete meaning of the original English scale clearly and accurately. Ten high-school students were then invited to evaluate the readability of the translated scale and some minor feedback was received, which was used to develop the final version of the Chinese-language SATAQ-4R. As a part of the research project investigating validity and reliability of exercise-related instruments among Chinese samples, this study was approved by the Ethics Committee of Shenzhen University in China (NO. PN-2022-00026).

Participants were informed that all data were kept confidential, and their responses were recorded anonymously. Demographic items were presented for completion first, followed by six self-reported scales including the SATAQ-4R, Drive for Muscularity Scale (DMS), Perceived Stress Scale (PSS), Eating Disorder Diagnostic Scale (EDDS), Body Image Acceptance and Action Questionnaire −5 (BIAAQ-5), Body Esteem Scale-Appearance (BES-appearance) and the Rosenberg Self-Esteem Scale (RSES). In addition, some participants voluntarily continued to the re-test of the SATAQ-4R about 1 week later. All data were collected *via* an online survey platform (Wenjuanxing). Three polygraph questions were inserted in the scales to rule out those participants who did not carefully read the items.

### Measures

Two gender-appropriate SATAQ-4Rs were used to assess the internationalization of appearance ideals and appearance-related sociocultural pressures from peers, families, media, and significant others (16). The SATAQ-4R-Female has 31 items within 7 sub-scales: (1) Internalization: thin/low body fat (four items, e.g., “I want my body to look very thin”); (2) Internalization: muscular (five items, e.g., “It is important for me to look muscular”); (3) Internalization: general attractiveness (six items, e.g., “I think a lot about my appearance”); (4) Pressures: family (four items, e.g., “I feel pressure from family members to look thinner”); (5) Pressures: peers (four items, e.g., “My peers encourage me to get thinner”); (6) Pressures: significant others (four items, e.g., “I feel pressure from significant others to improve my appearance”); and (7) Pressures: media (four items, e.g., “I feel pressure from the media to look in better shape”). Each item has 5 options of 1 (definitely disagree), 2 (mostly disagree), 3 (neither agree nor disagree), 4 (mostly agree), and 5 (definitely agree). Score on each sub-scale can be obtained as the sum of all items divided by the number of items, with higher mean scores indicating greater internalization or pressures. The SATAQ-4R-Male contains 28 items within 7 sub-scales/identical naming (see Supplementary Table 1).

The Drive for Muscularity Scale (DMS) was used to assess participants’ desire for muscularity and engagement in behaviors and attitudes to achieve a muscular physique (22). This scale consists of 15 items with two factors (e.g., “I wish I were more muscular,” “I think that my legs are not muscular enough”), with each item being rated on a 6-point response scale (0 = never to 6 = always). The score on each factor was calculated by the sum of all items divided by the number of all items, and a higher mean score indicates a greater level of motivation to acquire a muscular appearance. The DMS has been validated within Chinese female adults, demonstrating good internal consistency and validity (23). The Chinese-speaking DMS was used in this study, Cronbach’s alpha in the current sample was high with 0.83.

The Body Image Acceptance and Action Questionnaire −5 (BIAAQ-5) was used to assess participants’ flexible responses to body-related thoughts and feelings (24). This scale contains 5 items (e.g., “I shut down when I feel bad about my body shape or weight,” “Feeling fat causes problems in my life”) with each response ranging from 1 (never true) to 7 (always true). All items were reverse-coded. The score was calculated by the sum of all items, with higher sum scores indicating a higher level of body image flexibility. The BIAAQ-5 was validated among Chinese undergraduates, with good internal consistency and validity (25). The Chinese speaking BIAAQ-5 was used in this study, Cronbach’s alpha in the current sample was very high with 0.88.

One sub-scale (namely BES-appearance) of the Body Esteem Scale (BES) was used to assess an individual’s general feeling about one’s appearance (26) This sub-scale contains 10 positively or negatively worded items (e.g., “I’m pretty happy about the way I look,” “There are lots of things I’d change about my looks if I could”). The respondents indicate their degree of agreement with the questions on a 5-point Likert scale ranging from 0 (never) to 4 (always). The score was calculated by the sum of all items. The BES-appearance was validated among Chinese adolescents, with good internal consistency and validity (27). The Chinese-speaking BES-appearance was used in this study, Cronbach’s alpha of the BES-appearance in the current sample was high with 0.77.

The Perceived Stress Scale (PSS) was used to assess an individual’s stress level during last month (28). The PSS contains 10 items (e.g., “In the last month, how often have you felt nervous and stressed?,” “In the last month, how often have you felt that things were going your way?”). The respondents indicate their degree of agreement on a 5-point Likert scale ranging from 0 (never) to 4 (very often). The score is calculated by the sum of all items. PSS was validated among Chinese adolescents, with good internal consistency and validity (29). The Chinese-speaking PSS was used in this study, Cronbach’s alpha in the current sample was high with 0.83.

The Eating Disorder Diagnostic Scale (EDDS) is a brief self-reported scale for capturing eating disorder symptoms (30). The EDDS has 22 items, most of which are measured on a seven-point scale (0 = not at all to 6 = extremely), based on the past 3 months (e.g., “Over the past 3 months, have you felt fat?”). The EDDS also includes some Yes/No questions (e.g., “During these episodes of overeating and loss of control did you eat much more rapidly than normal? YES/NO”) and frequency assessments (e.g., “How many times per week on average over the past 3 months have you made yourself vomit to prevent weight gain or counteract the effects of eating?”). It yields a symptom-related composite score criteria for eating disorders. The EDDS had good reliability and validity in a large sample of male and female Hong Kong pupils (31). The Chinese-speaking EDDS was used in the current study, Cronbach’s alpha for the EDDS-5 was high of 0.87.

The Rosenberg Self-Esteem Scale (RSES) was used to assess global self-esteem and general feelings of self-worth. All 10 items are rated on a 4-point scale (1 = strongly disagree to 4 = strongly agree), with five items stating positive feeling and five items stating negative feeling. Score on items for negative feeling were firstly reversed and then add up to. The score was calculated by the sum of all items, and a higher sum score indicates a greater self-esteem. The Chinese-speaking RSES was translated and validated with good construct validity, internal consistency, and test–retest reliability (32). The Chinese-speaking RSES was used in this study, Cronbach’s alpha in the current sample was very high with 0.89.

### Statistical analysis

Data analysis was performed using Amos 28 (IBM SPSS, Chicago, United States). Given that the gender-appropriate scales were used across different countries and have been previously validated in a sample of Chinese adults, the female sample (*N* = 346) and the male sample (*N* = 344) were separately analyzed using the Confirmatory factor analysis (CFA) to evaluate internal consistency and convergent validity while maximum likelihood estimation was selected to test the seven-factor model presented in the original study (16). Model fit indices were used to determine if this seven-factor model was acceptable among Chinese adolescents in terms of gender: (1) Chi-square test *p* > 0.05; (2) RMSEA = 0.05 to 0.08 (reasonable fit); (3) CFI = 0.90 to 0.95 (reasonable fit); (4) SRMR<0.1. With regard to each sub-scale of the two gender-appropriate SATAQ-4R, its acceptable internal consistency was assessed using Cronbach’s alpha >0.70 (33). while convergent validity was tested using Pearson product–moment correlations, with r of 0.1 (small), 0.3 (medium), and 0.5 (large) (34); Specifically, correlations between sub-scales of the gender-appropriate SATAQ-4R and other variables (i.e., drive for muscularity, self-esteem, perceived stress, general appearance feeling and eating disorder symptoms) were examined for convergent validity. The test–retest reliability for all sub-scales of the SATAQ-4R were assessed using the intraclass correlation coefficients (ICC) in terms of gender-based sample. Its cutoff was rated as follows: poor: < 0.40; fair: ≥ 0.40 and ≤ 0.59, good: ≥ 0.60 and ≤ 0.74, and excellent: ≥ 0.75 and ≤ 1.00 (34, 35).

## Results

### Confirmatory factor analyses

**Figure 1:**
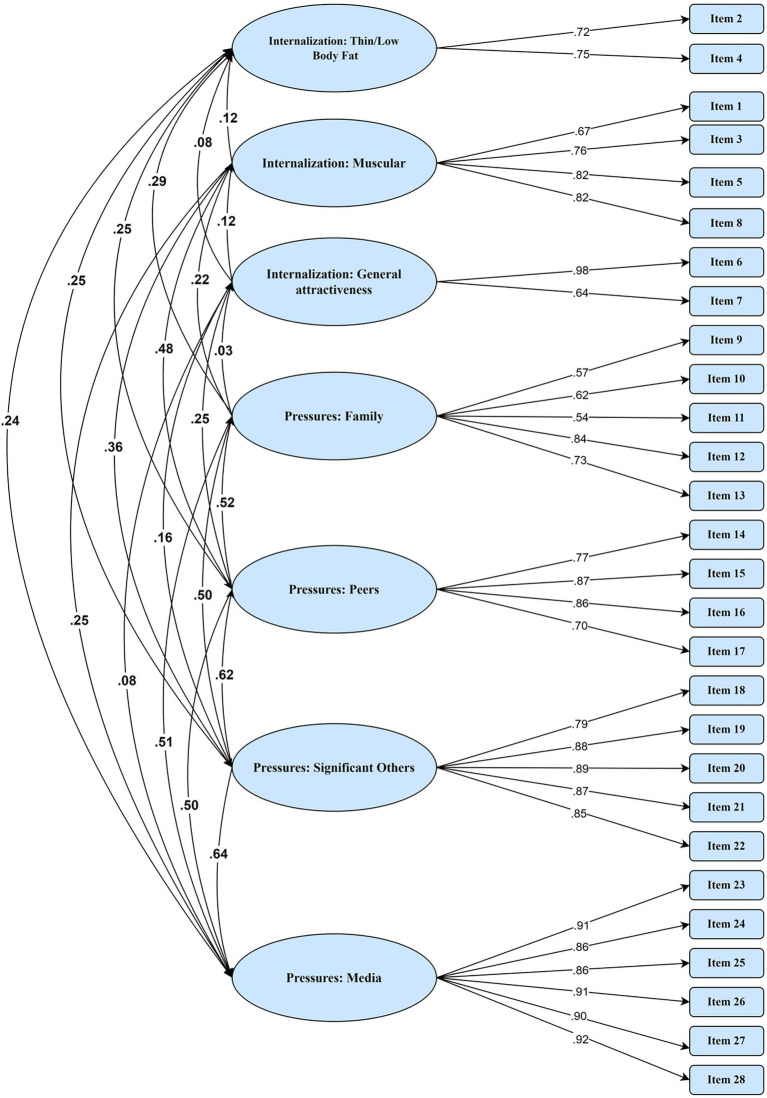
Confirmatory factor analysis for female adolescents.

**Figure 2:**
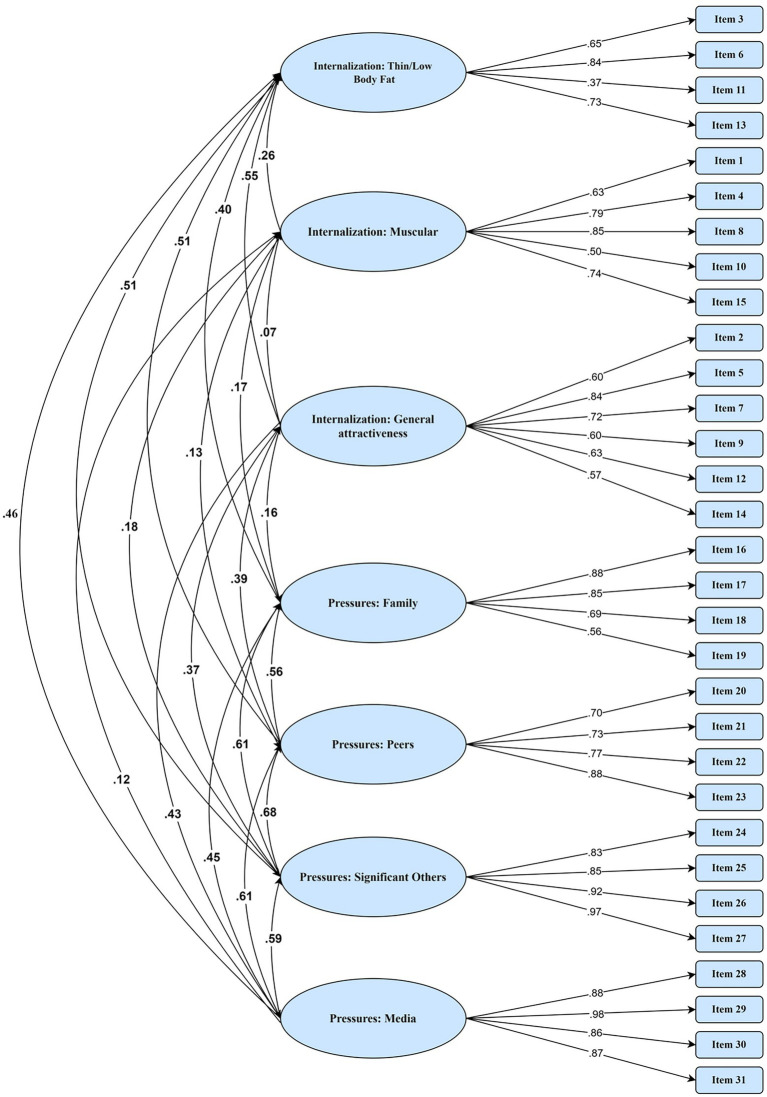
Confirmatory factor analysis for male adolescents.

For the SATAQ-4R-Females, descriptive statistics were calculated for all items. For each item, skew was lower than 2 and kurtosis was lower than 7, suggesting normal distribution of all items. All items loaded onto their original factor, ranging from 0.37 (Item 11) to 0.97 (Item 27; See Supplementary Table 1). The seven-factor model has a reasonable fit, with Chi-square = 1112.769 (*p* < 0.001), CFI = 0.91, RMSEA = 0.071, SRMR = 0.067 (Figure 1). For the SATAR-4R-Males, all items were loaded strongly on their intended factor, ranging from 0.54 to 0.98 (see Supplementary Table 2), generating an acceptable seven-factor model with Chi-square = 982.92 (*p* < 0.001), CFI = 0.91, RMSEA = 0.08, SRMR = 0.06 (Figure 2).

### Means, internal consistency, and intercorrelation between sub-scales

For the SATAQ-4R-Female, the mean score (ranging from 2.41 to 3.69) for sub-scales indicated a moderate level of ideal internalization and sociocultural pressure. The internal consistency for 7 subscales is rated as good, as indicated by Cronbach’s alpha =0.74 to 0.95 (see Supplementary Table 1). In addition, intercorrelations between every two sub-scales were significantly positive, except for the correlation between muscularity and general attractiveness (*r* = 0.07, *p* > 0.05) in terms of internalization (Table 2).

For the SATAQ-4R-Male, the mean score for each sub-scale indicated that adolescent boys experienced a moderate level of appearance ideal internalization (ranging from 2.47 to 3.32) and low to moderate level of sociocultural pressures (ranging from 2.37 to 2.76). The internal consistency of the seven sub scales is rated as good (Cronbach’s alpha =0.70 to 0.96; see Supplementary Table 2). There were three non-significant correlations (i) between thin/low body fat-Internalization and general attractiveness-Internalization; (ii) general attractiveness-Internalization and family-Pressures and (iii) general attractiveness-Internalization and media-Pressures (See Table 3).

**Table 3:** Means and inter-correlations between the Chinese SATAQ-4R Male Subscales (*N* = 335).

	Mean (SD)	1	2	3	4	5	6	7
Internalization: Thin/Low Body Fat	2.47 (0.94)							
Internalization: Muscular	3.15 (0.86)	0.12*						
Internalization: General attractiveness	3.32 (0.87)	0.08	0.12*					
Pressures: Family	2.37 (0.77)	0.29**	0.22**	0.03				
Pressures: Peers	2.76 (0.93)	0.25**	0.48**	0.25**	0.52**			
Pressures: significant others	2.59 (0.96)	0.25**	0.36**	0.16**	0.50**	0.62**		
Pressures: Media	2.29 (0.96)	0.24**	0.25**	0.08	0.51**	0.50**	0.64**	

SD = standard deviation, * = *p* < 0.05, ** = *p* < 0.01.

### Convergent validity

For the SATAQ-4R-Female, except for muscularity, other sub-scales were: (1) positively (lightly to moderately) associated with eating disorder symptoms; (2) negatively (lightly to moderately) associated with body image flexibility; (3) negatively (lightly) associated with self-esteem. The Internalization: Muscular subscale demonstrated large associations with the variable drive for muscularity, and small associations with eating disorder symptomatology, but was not significantly associated with body image flexibility and self-esteem. The Pressures subscales presented small to medium positive associations with measures of perceived stress (see Table 4). For the SATAQ-4R-Male, the Internalization: Thin/Low Body Fat subscale exhibited a small positive correlation with body image flexibility. The Internalization: Muscular subscale exhibited large negative associations with drive for muscularity (assessed *via* DMS). The Internalization: General Attractiveness presented medium associations with body image flexibility. The Pressure: Peers and Pressure: Significant Others subscales presented small associations with measures of perceived stress (see Table 4).

**Table 4:** Correlations between the Chinese SATAQ-4R subscales and convergent measures. (Female: *N* = 344; Male: *N* = 335).

	Internalization						Pressures							
	Thin/ Low Body Fat		Muscular		General attractiveness		Family		Peers		Significant others		Media	
	F	M	F	M	F	M	F	M	F	M	F	M	F	M
DFM	−0.19**	−0.03 ^n.s^	−0.56**	−0.67**	−0.11 ^n.s^	−0.24**	−0.21**	−0.21**	−0.11*	−0.51**	−0.14**	−0.42**	−0.07 ^n.s^	−0.29**
BIAAQ	0.53**	0.29**	0.10 ^n.s^	0.19**	0.41**	0.17**	0.43**	0.30**.	0.51**	0.35**	0.49**	0.35**	0.52**	0.38**
BES-APP	.-0.35**	−0.19**	−0.06	−0.05 ^n.s^	−0.31**	−0.08 ^n.s^	.-0.26**	−0.24**	−0.36**	−0.31**	0.28**	−0.26**	−0.30**	−0.21**
PSS	0.29**	0.09 ^n.s^	0.07 ^n.s^	0.26**	0.34**	0.05 n.s	0.18**	0.05 n.s	0.29**	0.17**	0.26**	0.24**	0.30**	0.10 ^n.s^
EDDS	0.43**	0.19**	0.15**	0.08 ^n.s^	0.33**	0.13*	0.31**	0.16**	0.33**	0.22**	0.34**	0.25**	0.33**	0.31**
RESE	−0.22**	−0.17**	−0.06	0.09 ^n.s^	−0.24**	−0.08 ^n.s^	−0.15**	−0.10 n.s	−0.23**	−0.15	−0.22**	−0.13*	−0.20**	−0.04

DFM = Drive for Muscularity Scale, BIAAQ = Body Image-Acceptance and Action Questionnaire, BES-APP = Body Esteem Scale - Appearance, PSS = Perceived Stress Scale, EDDS = Eating Disorder Diagnostic Scale, RESE = Rosenberg Self-Esteem Scale, F = female, M = male, * = *p* < 0.05, ** = *p* < 0.01, n.s = no significance.

## Discussion

The current study evaluated the reliability and validity of the gender appropriate SATAQ-4R within the sample of Chinese adolescent girls and boys. Specifically, each of the subscales demonstrated good internal consistency and acceptable test–retest reliability. In addition, our results also confirmed its convergent validity of the two different gender-appropriate scales. Also, our study has clarified the structure of the SATAQ-4R among age the group between 15 and 17, which was not specifically validated in previous research. In the following, we will discuss our findings with further details.

### Factor structure

Generally, this study provided evidence that the gender-specific SATAQ-4Rs are valid and reliable instruments that can be applied in samples of Chinese female and male adolescents. With respect to the factor structure, the results of the present study support the findings of the original study with US college students with a mean age of 20 years (16) and other culturally adapted scales among Italian adults with a mean age of 20 years (18) and among female college students in Turkey (36) in terms of gender-specific SATAQ-4R. On the other hand, other previous validation studies had also generated different factor structures. For example, another validation study demonstrated two 5-factor scales among Brazilian children aged 7–11 years (37) while a 6-factor structure for 225 male participants and a 4-factor structure for 308 male participants were observed among Chinese adults aged 18 to 68 years – of note, this non-validation study only reported Cronbach’s a and EFA of the SATAQ-4R (but no CFA-related fit indices and test–retest reliability) (17). Taking the above-presented findings into account, it seems reasonable to hypothesize that age is a factor that may alter the factor structure of the SATAQ-4R. This assumption is supported by the fact that high-school students in China (mean age of 16 years) being studied in the current trial and western adults (mean age of 20 years) show a comparable factor structure (16), whereas in samples of Brazilian children and Chinese adults (38) (mean age of 9 years and 30 years, respectively) age-related effects may contribute to the differences being observed concerning the factor structure (6-factor and 4-factor structure, respectively). To verify or refute the assumption that the factor structure of the SATAQ-4R varies as a function of age, more high-quality studies following the standard validation procedures should be conducted among various age groups and health conditions.

### Internal consistency

Overall, the results emerging from the female sample demonstrated a good internal consistency of the SATAQ-4R scale. Of note, muscularity-Internalization did not show a strong inter-correlation with other subscales of SATAQ-4R, ranging from 0.12 (media-Pressure) to 0.26 (significant others-Pressure). This finding is in line with the previous studies conducted in a different sociocultural context (e.g., western societies such as the United States) or within Asian culture (16, 18, 37, 39). In addition, the correlation between muscularity-Internalization and general attractiveness-Internalization was not observed among female adolescents. Such a finding was expected as previous research has shown that muscularity is not the sociocultural feminine standard of attractiveness in the Chinese culture (40). Moreover, there is evidence in the literature suggesting that Chinese females have a lower motivation to exercise and a lower level of internalization for muscularity comparison to females in western societies (41, 42). The sociocultural ideal of female beauty that is commonly mentioned in the Chinese culture includes attributes such as being tall and curvy (40). Taken together, muscularity does not play an important role in the sociocultural expectation of the ideal body image of Chinese females which might explain the non-significant correlation between these two subscales. Male-related results in the present study demonstrated acceptable internal consistency. In contrast to studies conducted within the Western culture, no correlation was observed between the subscales Internalization: general attractiveness and internalization of thin ideal, pressure from family, or pressure from media. However, this result is in line with the previous research conducted within Chinese adults (17), suggesting that males in China are less likely to be encouraged to evaluate their appearance by their family or on social media compared to females.

### Convergent validity

Consistent with our hypotheses (male participants), all the pressure-related subscales and Thin/low body fat-Internalization subscale demonstrated small to medium positive correlation with eating disordered symptoms and medium negative correlations with body image acceptances/flexibility, which is in line with findings in samples of American and Italian men samples (16, 18). This agrees with the tripartite model suggesting that perceived sociocultural pressures and internalization of thinness contribute to negative eating and body image outcomes. With PSS, only two pressure-related subscales, peer-Pressures, and significant others-Pressures, demonstrated medium to large positive associations with perceived stress levels. Taken together, it appears that elevated perceived stress levels, increased eating disorder symptoms, and decreased body image acceptance may be caused by the fact that Asian people including Chinese are more concerned about (good) physical appearance (through very strict eating habits) as they are linked to career success in the modern society– naturally developing pressures from peers and significant others. In addition, only significant others-Pressures exhibited a medium negative association with self-esteem, which may be attributed to the unique cultural context of China a great emphasis on collectivism (43). Such Chinese cultural collectivism linked to perceived stress could lead to reduced self-esteem.

The female version of the SATAQ-4R has established good convergent validity among the sample of Chinese adolescent girls. As we have mentioned above, muscularity is more relevant to males than females. Chinese females have a lower motivation for muscularity due to different beauty standards and sociocultural impact in the Chinese culture, so that it seems reasonable to suggest that the absence of statistically significant correlations between internalization of muscularity and body image flexibility, body esteem, perceived stress, or self-esteem is related to the former (i.e., beauty ideal in China). However, a small correlation was observed between internalization of muscularity and eating disorder symptoms. Furthermore, muscularity-Internalization demonstrated a large positive association with a drive for muscularity, but it was not associated with eating disorder symptoms and self-esteem. Such results are different from previous studies focusing on American and Italian males (16, 18). Unlike Western society which has an inveterate link between muscularity and masculinity, Chinese adolescent boys seem to have less exposure to exaggerated muscular body ideals and may not attach muscularity to individual popularity on campus (44), therefore, whether being muscular or not seems not to influence their eating behavior or self-esteem (i.e., based on the absence of correlations in the empirical data of the current study).

General attractiveness-Internalization demonstrated a small positive association with eating disorder symptoms, but it failed to correlate with general appearance feelings. It is likely that the items of BES-appearance were perceived to assess facial attractiveness as opposed to overall body figure in the general-attractiveness-Internalization based on their respective contexts. Therefore, these two scales might evaluate two distinctive constructs due to their contextual differences, which might be the reason why they did not reach convergence.

The validated version of SATAQ-4R would be valuable for advancing psycho-social health and well-being among Chinese adolescents emphasizing gender-specific challenges associated with body dissatisfaction. Given a high burden of body image disturbances and associated mental health crises in this population (45, 46), the findings of this study will allow researchers to use robust measurement approaches to assess and address such psycho-social problems. Also, future research evaluating the relevance of the current scale and other psychometric instruments may benefit from the findings of the current study in the given population context. The use of validated instruments is critical for evaluating and treating mental and behavioral health problems, which necessitates further research promoting context-specific estimations of psycho-social health problems for maximizing health and social outcomes across different populations.

### Study limitations

This study also has some limitations that need to be acknowledged and considered when interpreting our findings. The test–retest reliability assessment in the current study is based on rather small samples which, in turn, might somewhat limit the generalizability of these findings, although our results are encouraging as they suggest a good test–retest reliability of both versions of the SATAQ-4R (i.e., female and male versions). However, to address the latter limitation, future studies should seek to evaluate the test–retest reliability of both versions of the SATAQ-4R in larger samples with different age groups.

## Conclusion

There is evidence in the literature suggesting that mental health conditions including eating disorders generally emerge among Chinese adolescents and young adults. Considering the social and health burden resulting from these mental health problems (mainly depressive disorders and eating disorders), the examination of sociocultural factors that influence the physical and mental health of Chinese adolescents is urgently needed. In this context, the findings of the current studies which provide evidence that the SATAQ-4R-Male and SATAQ-4R-Female have good psychometric properties to assess appearance-ideal internalization and appearance pressures in Chinese adolescent girls and boys, pave the way for future investigations on the appearance-ideal internalization among Chinese adolescents. The SATAQ-4R may also be used in future studies to test various theoretical models of the etiology of disordered eating, such as the tripartite influence model, within Chinese adolescents. Additionally, eating disorder treatments mainly aim to reduce the internalization of appearance ideals and address negative appearance-related pressures from sociocultural groups. Therefore, clinicians and researchers may consider the inclusion of the current measure (i.e., translated and validated version of the SATAQ-4R) to identify mechanisms of change in eating disorder prevention with patients or clinical research trials.

## Data availability statement

The raw data supporting the conclusions of this article will be made available by the authors, without undue reservation.

## Ethics statement

The studies involving human participants were reviewed and approved by the Ethics Committee of Shenzhen University in China (No. PN-2022-00026). As a part of the research project investigating validity and reliability of exercise-related instruments among Chinese samples. Written informed consent to participate in this study was provided by the participants’ legal guardian/next of kin.

## Author contributions

HH, ZL, HX, and LZ: conceptualization and formal analysis. HH, ZL, and HX: data curation, investigation, methodology, software, and writing – original draft. LZ: project administration, resources, and validation. JK and LZ: supervision. HH, ZL, HX, FH, JK, EC, AT, AY, JS, MH, AK, TG, and LZ: writing – review and editing. All authors contributed to the article and approved the submitted version.

## Funding

This study was partially supported by Start-up Research Grant of Shenzhen University (20200807163056003) and Start-Up Research Grant (Peacock Plan: 20191105534C), the Natural Science Foundation of Guangdong Province of China (Grant no. 2018A030307002), the National Nature Science Foundation of China (Grant no. 31871115), and funding from the Shenzhen Humanities & Social Sciences Key Research Bases of the Center for Mental Health, Shenzhen University.

## Conflict of interest

The authors declare that the research was conducted in the absence of any commercial or financial relationships that could be construed as a potential conflict of interest.

## Publisher’s note

All claims expressed in this article are solely those of the authors and do not necessarily represent those of their affiliated organizations, or those of the publisher, the editors and the reviewers. Any product that may be evaluated in this article, or claim that may be made by its manufacturer, is not guaranteed or endorsed by the publisher.
