# Supplementary material for: Validation of sociocultural attitudes towards appearance questionnaire and its associations with body-related outcomes and eating disorders among Chinese adolescents
Source: Front Psychiatry. 2023 Mar 13;14:1088769. doi: 10.3389/fpsyt.2023.1088769 (PMC10041934; doi:10.3389/fpsyt.2023.1088769)
Supplement: Supplementary file 1 [file Table_1.DOCX]

**Supplementary materials**

Table 1. Chinese SATAQ-4R Female Factor Loading and Internal Consistency.

| Items | Internalization | | | Pressure | | | |
| --- | --- | --- | --- | --- | --- | --- | --- |
|  | Thin/Low  Body Fat | Muscular | General  Attractiveness | Family | Peers | Significant  Others | Media |
| Item 1 |  | .63 |  |  |  |  |  |
| Item 2 |  |  | .60 |  |  |  |  |
| Item 3 | .65 |  |  |  |  |  |  |
| Item 4 |  | .79 |  |  |  |  |  |
| Item 5 |  |  | .84 |  |  |  |  |
| Item 6 | .84 |  |  |  |  |  |  |
| Item 7 |  |  | .72 |  |  |  |  |
| Item 8 |  | .85 |  |  |  |  |  |
| Item 9 |  |  | .60 |  |  |  |  |
| Item 10 |  | .50 |  |  |  |  |  |
| Item 11 | .37 |  |  |  |  |  |  |
| Item 12 |  |  | .63 |  |  |  |  |
| Item 13 | .73 |  |  |  |  |  |  |
| Item 14 |  |  | .57 |  |  |  |  |
| Item 15 |  | .74 |  |  |  |  |  |
| Item 16 |  |  |  | .88 |  |  |  |
| Item 17 |  |  |  | .85 |  |  |  |
| Item 18 |  |  |  | .69 |  |  |  |
| Item 19 |  |  |  | .56 |  |  |  |
| Item 20 |  |  |  |  | .70 |  |  |
| Item 21 |  |  |  |  | .73 |  |  |
| Item 22 |  |  |  |  | .77 |  |  |
| Item 23 |  |  |  |  | .88 |  |  |
| Item 24 |  |  |  |  |  | .83 |  |
| Item 25 |  |  |  |  |  | .85 |  |
| Item 26 |  |  |  |  |  | .92 |  |
| Item 27 |  |  |  |  |  | .97 |  |
| Item 28 |  |  |  |  |  |  | .88 |
| Item 29 |  |  |  |  |  |  | .98 |
| Item 30 |  |  |  |  |  |  | .86 |
| Item 31 |  |  |  |  |  |  | .87 |
| Cronbach’s alpha | .74 | .82 | .83 | .85 | .87 | .95 | .95 |

Table 2. Chinese SATAQ-4R Male Factor Loadings and Internal Consistency.

| Items | Internalization | | | Pressure | | | |
| --- | --- | --- | --- | --- | --- | --- | --- |
|  | Thin/Low  Body Fat | Muscular | General  Attractiveness | Family | Peers | Significant  Others | Media |
| Item 1 |  | .67 |  |  |  |  |  |
| Item 2 | .72 |  |  |  |  |  |  |
| Item 3 |  | .76 |  |  |  |  |  |
| Item 4 | .75 |  |  |  |  |  |  |
| Item 5 |  | .82 |  |  |  |  |  |
| Item 6 |  |  | .98 |  |  |  |  |
| Item 7 |  |  | .64 |  |  |  |  |
| Item 8 |  | .82 |  |  |  |  |  |
| Item 9 |  |  |  | .57 |  |  |  |
| Item 10 |  |  |  | .62 |  |  |  |
| Item 11 |  |  |  | .54 |  |  |  |
| Item 12 |  |  |  | .84 |  |  |  |
| Item 13 |  |  |  | .73 |  |  |  |
| Item 14 |  |  |  |  | .77 |  |  |
| Item 15 |  |  |  |  | .87 |  |  |
| Item 16 |  |  |  |  | .86 |  |  |
| Item 17 |  |  |  |  | .70 |  |  |
| Item 18 |  |  |  |  |  | .79 |  |
| Item 19 |  |  |  |  |  | .88 |  |
| Item 20 |  |  |  |  |  | .89 |  |
| Item 21 |  |  |  |  |  | .87 |  |
| Item 22 |  |  |  |  |  | .85 |  |
| Item 23 |  |  |  |  |  |  | .91 |
| Item 24 |  |  |  |  |  |  | .86 |
| Item 25 |  |  |  |  |  |  | .86 |
| Item 26 |  |  |  |  |  |  | .91 |
| Item 27 |  |  |  |  |  |  | .90 |
| Item 28 |  |  |  |  |  |  | .92 |
| Cronbach’s alpha | .70 | .85 | .77 | .80 | .88 | .94 | .96 |
